# Gi4SaveLife: a community-led model for sustainable voluntary blood donation in rural Sierra Leone

**DOI:** 10.1136/bmjgh-2026-023974

**Published:** 2026-06-15

**Authors:** Cristina Fernandez Turienzo, Lucy November, Mohamed Turay, Philemon Kamara, Rick K Lassie, John S Kamara, Mohamed K Kamara, Mangenda Kamara, Mariatu Duraman, Katy Kuhrt, Andrew J M Leather, Sahr Gevao, Prince T Williams

**Affiliations:** 1 Department of Women & Children’s Health, School of Life Course & Population Health Sciences, Faculty of Life Sciences & Medicine, King’s College London, London, UK; 2 Lifeline Nehemiah Projects, Freetown, Sierra Leone; 3 University of Sierra Leone, Freetown, Sierra Leone; 4 Koidu Government Hospital, Koidu, Sierra Leone; 5 Department of Population Health Sciences, School of Life Course & Population Health Sciences, Faculty of Life Sciences & Medicine, King’s College London, London, UK

**Keywords:** Africa South of the Sahara, Global Health, Health systems, Public Health

## Abstract

Sierra Leone faces critical blood shortages reliant on family replacement and paid donors, contributing to high maternal mortality. Traditional top-down blood drives have failed to build sustainable voluntary donor bases due to mistrust and logistical barriers. We developed 'Gi4SaveLife’, a community-led model to foster local ownership and sustainable donation. Implemented in Kono and Kenema districts, this quality improvement initiative established community 'hubs’ led by local volunteers responsible for peer-to-peer education and mobilisation. We conducted a baseline survey to identify barriers, followed by monthly blood drives using non-monetary incentives. Data on collection volumes, donor demographics and costs were tracked over 8 months. The initiative collected 539 units from 376 unique donors, moving from zero voluntary donations at baseline to a consistent supply. Crucially, 43% (161) were repeat donations, indicating a committed donor pool. The cost per unit decreased by 40% compared with standard NGO-led models. Key motivators included altruism (71%) and social recognition (52%). Gi4SaveLife demonstrates that a community-led approach, grounded in ownership and trust, can establish a cost-effective, sustainable pipeline of voluntary donors in low-resource settings. This model offers a scalable framework to bolster national blood transfusion services by bridging the gap between communities and health facilities.

**SUMMARY BOX**

- Sierra Leone faces severe shortages of voluntary non-remunerated blood donors, relying heavily on family replacement and paid donors.
- Traditional, top-down blood drives often fail to build trust or long-term donor commitment in rural communities.
- Gi4SaveLife, a community-led 'hub’ model in rural Sierra Leone, successfully generated a consistent supply of voluntary blood with a 43% repeat donation rate, while reducing the cost per unit by 40% compared with standard hospital-led drives.
- Community ownership is not just an ethical imperative but a practical mechanism for sustainability and cost-efficiency in blood donor mobilisation.
- This model provides a scalable strategy that can support national blood transfusion services by managing donor recruitment at the community level in similar contexts.

## Introduction

 Access to a safe and sufficient blood supply is a non-negotiable component of a functioning health system, critical for emergency medicine, surgery and maternal and child health.^1^ Yet, the global distribution of this life-saving resource is sharply unequal. Low- and middle-income countries (LMICs), which bear the highest burden of disease and injury, face the most severe shortages.^2^ The WHO has long advocated for all countries to move towards 100% voluntary non-remunerated blood donation (VNRBD) as it is the foundation of a safe, sustainable national blood supply^3^, and although high-income countries have largely achieved this, many nations in sub-Saharan Africa remain heavily dependent on family replacement and paid donors.^4^

Sierra Leone is one of the countries that faces this challenge. With a very high maternal mortality ratio despite recent improvements, postpartum haemorrhage remains a leading cause of death.^5^ The inability to access blood quickly is a major contributing factor. Nationally, only 15% of the required blood supply is met through VNRBD, leading to critical shortages and preventable loss of life.^6^ The remainder is sourced from paid blood donors who congregate outside hospitals and family members in moments of crisis, a system that introduces dangerous delays, creates financial hardship and fails to maintain a safe, adequate and preemptive blood stock.^4^ For adolescent girls and unsupported pregnant women, who are at high risk during childbirth and often lack family support, this system is particularly risky.^7^

The barriers to VNRBD in Sierra Leone are complex and deeply rooted, reflecting well-known challenges across sub-Saharan Africa, including a lack of public awareness, logistical challenges and cultural myths and fears (for example, that donating blood leads to illness or infertility or that donated blood may be used for witchcraft).^8 9^ Traditional blood donation campaigns, often organised externally by health workers, have struggled to make lasting inroads. These top-down 'blood drive’ models frequently lack the sustained community engagement needed to build trust, dispel misinformation and foster a culture of regular donation. Characterised by brief site visits to collect blood and immediately depart, such approaches fail to establish the sense of community ownership that underpins sustainability.^10^

Recognising these systemic failures, Lifeline Nehemiah Projects (LNP), a Sierra Leonean community-based organisation, developed 'Gi4SaveLife’, building on their experience and principles from the 2YoungLives community-led mentoring model for adolescent pregnant girls that reduced maternal and perinatal mortality composite by 48%.^11^ LNP planned a community-led model to fundamentally invert the traditional model; instead of taking a top-down approach, Gi4SaveLife is an intervention built from within the community itself. Its rationale is grounded in the theory that sustainable change requires local ownership, trusted champions and solutions tailored to the specific cultural context.^10 12^ We aim to describe the implementation and impact of the Gi4SaveLife pilot in a rural district of Sierra Leone and determine if this community-led model could create a sustainable mechanism for donor mobilisation to support the national blood donation system, thereby increasing the availability of safe blood, reducing a critical delay in maternal healthcare and ultimately reducing preventable maternal deaths.

## Gi4SaveLife: context and intervention

The Gi4SaveLife initiative was implemented in areas within the rural districts of Kono and Kenema in the Eastern Province of Sierra Leone from April 2022 to May 2023. The partner institution was Koidu Government Hospital (KGH), the primary government referral hospital in the area. KGH has a functional blood bank, renovated in 2016, with capacity for safe screening and storage. However, prior to the intervention, its blood supply was critically low and entirely dependent on family replacement and paid donations. In January 2022, for example, 117 units were transfused to maternity patients, none of which came from voluntary donors. The hospital management and blood bank team identified the absence of voluntary donors as the single greatest limiting factor to providing timely transfusions. Three targeted communities were selected in consultation with local leaders. Gi4SaveLife was designed as a community-led strategy, using a pre-post design to assess its impact and SQUIRE 2.0 reporting guidelines.^13^

The Gi4SaveLife intervention was a multi-component, adaptive initiative centred on community ownership and empowerment:

### Community engagement and involvement

The foundational activity was a deep and sustained engagement with community structures. The LNP team, all Sierra Leoneans, held initial meetings with town chiefs, women’s leaders, youth leaders, religious figures, and other key influencers from the three target communities. This was not simply to gain permission but to build a genuine partnership and co-design the initiative.

### Baseline needs assessment

To ensure the intervention was context-specific, we conducted a local baseline assessment; this included a 137-participant survey to quantify local knowledge, attitudes, and practices related to blood donation. Administered face-to-face by local team members to ensure accessibility for non-literate participants, the survey systematically identified prevalent myths and barriers (eg, 23% believed donated blood could be used for witchcraft; 22% feared it caused infertility). See details in online supplemental file S1. This assessment directly informed the educational content.

### The 'Hub’ model

The core of the intervention was the development of five community 'hubs’, small, localised teams of volunteers responsible for driving the initiative. Each hub consisted of a leader and five members, nominated by their own communities based on their social standing and influence, particularly among young people. The 25 hub members became the trusted, local face of the project. They received a 2-day training workshop by LNP, focused on blood donation facts, communication skills and strategies for addressing the specific myths identified in the baseline survey.

### Donor recruitment and support

Hub members were responsible for continuous, peer-to-peer sensitisation and donor recruitment within their social networks, engaging farmers, students, motorbike riders and traders. Their role extended beyond recruitment; they provided support to donors during the drives and conducted follow-ups to ensure their well-being, in line with the organisation’s ethos of building caring community networks.

### Blood drives and non-monetary incentives

Monthly blood drives were conducted in accessible community locations. A key principle, aligned with WHO recommendations, was the avoidance of cash incentives. Instead, we tested a bundle of non-monetary motivators that included providing food and drinks at the drives, distributing branded wristbands to foster a sense of identity and pride, and organising social events like community football matches for donors.

### Publicity and mass communication

To amplify the message, we used local media channels, including popular radio stations (Eastern Radio and Radio Maria) and a television feature.^14 15^ These broadcasts featured LNP leaders and health professionals discussing the life-saving importance of voluntary donation and directly addressing common misconceptions.

## Methods of evaluation

The primary outcome was the number of units of blood collected per drive and cumulatively over the project period. We also tracked the number of unique donors and, importantly, the number of repeat donors, which served as a proxy for the sustainability of the donor base. We monitored the activities of the hubs, the content of training sessions, and the reach of media campaigns. An online donor survey (Qualtrics), administered after the fifth drive with face-to-face completion options, was used to understand donor experiences and motivations. We monitored for any unintended negative consequences, such as community conflict or dissatisfaction with the non-cash incentive model. The project’s potential for cost-effectiveness was analysed by calculating the cost per unit of blood collected at different phases of the project cycle.

Data on those outcome and process measures were collected by the nurse and/or project assistant on a MS Excel file (blood collection volume, donor numbers, costs) or via Qualtrics (self-administered survey). Data were analysed using descriptive statistics in MS Excel which we used to run charts to track the number of units collected over time. Data from the donor survey were analysed to determine the frequency of different motivators. The analysis focused on demonstrating improvement over time and understanding the mechanisms driving the observed changes.

The project was conducted as a quality implementation initiative in partnership with the Koidu Government Hospital and local community leadership. As such, formal external ethics review was not required under local guidelines. However, the project adhered to strict ethical principles. All survey participants provided informed consent. Donor confidentiality was paramount, especially regarding the results of blood screening for infections like HIV. The process was designed to be non-coercive, and all donations were entirely voluntary.

## Key findings

The Gi4SaveLife initiative demonstrated substantial success in achieving its primary goals in the targeted rural communities. Over the course of 8 monthly blood drives from July 2022 to May 2023, a total of 539 units of blood were collected, exceeding the project target of 510 units. These donations came from 376 unique individual donors. A key indicator of the project’s success in building a sustainable donor culture was the rate of repeat donations. Of the total donations, 161 were from repeat donors, demonstrating a significant shift from one-time, crisis-driven behaviour to regular, altruistic giving. The final blood drive in May 2023 saw the highest turnout, with 83 units collected, of which 55 (66%) were from repeat donors. A consistent and high level of collection was found across the drives, with numbers remaining strong even after initial financial stipends for hub members were phased out for the last two drives as part of the sustainability plan. The run chart of donations over time is shown in table 1.

**Table 1:** Run chart of donations over time

Date of blood drive	Total donors (n predicted)	First time donors (n predicted)	Repeat donors (n predicted)
07/07/2022	58 (50)	58 (50)	
26/08/2022	68 (50)	68 (50)	
04/11/2022	48 (50)	43 (50)	4 (0)
25/11/2022	57 (80)	51 (50)	6 (30)
16/12/2022	65 (80)	56 (50)	8 (30)
27/01/2023	80 (80)	72 (50)	8 (30)
18/03/2023	80 (60)	0 (0)	80 (60)
12/05/2023	83 (60)	28 (0)	55 (60)
Total	539 (510)	376 (300)	161 (210)

The donor survey provided critical insights into the drivers of this success. When asked to select their top reasons for donating, the responses covered a range of motives: purely altruistic (71% 'desire to save lives’); social recognition and positive identity (52% stated they 'enjoyed being known as a blood donor’); a form of health insurance (49% agreed with 'if I need blood or my family member needs blood 1 day, we will get it’); a direct health benefit (48% valued the opportunity to have a confidential blood test for HIV and other diseases); and a means of obtaining an immediate non-cash incentive (35% cited the provision of food at the drive as a motivator). See online supplemental file S2 for details.

The initiative also proved to be a potential cost-effective model. The initial setup phase, which included intensive community engagement and training, represented a front-loaded investment. As the project progressed and moved to a volunteer-led sustainability model, the cost per unit of blood collected fell dramatically. In the final, sustainable phase, the cost per unit was SLE 131 (£6 at that time), which was 40% lower than the estimated cost of SLE 225 (£10 at that time) per unit for existing NGO-funded, hospital-led models (online supplemental file S3). This demonstrates a clear value-for-money proposition.

Perhaps the most significant result was the culture shift within the communities. Anecdotal evidence, such as the story of a senior stakeholder who initially opposed the project on religious grounds but became a devoted advocate after his own family member’s life was saved with the donated blood, illustrated the power of the model to change deeply held mindsets. The project successfully reframed blood donation from a feared medical procedure into a valued, proactive and life-affirming act of community pride.

## Discussion

This quality improvement initiative demonstrates that a community-led, context-specific model can successfully establish a sustainable pipeline of voluntary donors in a low-resource setting, directly addressing a critical bottleneck in the healthcare system. The success of Gi4SaveLife offers important lessons for blood service programmes across Sub-Saharan Africa and other LMICs. The core strength of this intervention lies in its departure from the traditional, community-driven public health campaign. By vesting ownership, responsibility and social capital within the community itself, the project fostered a level of trust and resonance that top-down approaches rarely achieve.^10^ The 'hub’ volunteers were not outsiders; they were known and respected members of their own communities, and this allowed them to act as effective and credible agents of change, capable of navigating complex social dynamics and addressing misinformation in a culturally sensitive manner. This aligns with a growing body of evidence emphasising the critical role of community engagement in the success of global health interventions.^12 16 17^

Our findings on donor motivation reinforce that altruism is a powerful driver, but this coexists with other important factors. The desire for social recognition, the assurance of future access to blood and the tangible benefit of health screening were all significant motivators. This suggests that the most effective recruitment strategies are multi-faceted, appealing to a combination of intrinsic (doing good), reciprocal (community benefit) and personal (health checks, food) incentives.^18^ The successful phasing out of small financial stipends for volunteer hub members without a drop in engagement and donation rates is a particularly important finding, providing strong evidence for sustainability.^3^

The project also highlights the importance of bridging the gap between communities and the formal health system. While communities can drive demand and recruitment, this must be met by a functioning, government-led technical system for testing, cold-chain storage and distribution. This ‘meso-level’ work, at the interface of community and clinic, is often overlooked but is critical for ensuring that supply-side improvements (more blood) are met with effective demand-side integration (better hospital processes).^19 20^

While implemented in one district, the core mechanisms, local ownership and non-monetary trust-building are likely transferable to other low-resource settings facing similar trust deficits. The success was also heavily dependent on the strong existing reputation and community ties of the implementing partner, LNP. Replicating the model would require identifying or cultivating similarly trusted local organisations and ensuring integration with national blood transfusion guidelines and quality assurance protocols. Finally, while we demonstrated a decrease in the cost per unit, a full economic evaluation was beyond the scope of this project.

The Gi4SaveLife initiative offers a powerful, replicable model for addressing chronic blood shortages that challenge many health systems in LMICs by placing communities at the centre of change. Sustainable progress requires shifting investment from episodic, externally driven blood drives to long-term support for local civil society organisations working in coordination with national health authorities. This approach is more than a logistical fix; it is a transformative social process that strengthens community cohesion, dispels fear with knowledge and embeds a culture of shared responsibility for health.

## Supplementary material

10.1136/bmjgh-2026-023974 online supplemental file 1

## Data Availability

All data relevant to the study are included in the article or uploaded as supplementary information.
