# Supplementary material for: Gi4SaveLife: a community-led model for sustainable voluntary blood donation in rural Sierra Leone
Source: BMJ Glob Health. 2026 Jun 15;11(6):e023974. doi: 10.1136/bmjgh-2026-023974 (PMC13289003; doi:10.1136/bmjgh-2026-023974)
Supplement: online supplemental file 1 [file bmjgh-11-6-s001.docx]

**Supplementary material**

**Table S1 Initial community survey**

| **Survey question** | **Yes (%)** | **No (%)** |
| --- | --- | --- |
| Is all blood tested to ensure it is free from any infection? | 81 | 19 |
| Do you believe there is a risk of spreading disease through blood donation? | 44 | 56 |
| Do you think you can catch infections by donating blood? | 30 | 70 |
| If a person is anaemic, needing blood, do you associate that with witchcraft? | 14 | 86 |
| Do you believe that donated blood can be used for witchcraft? | 23 | 77 |
| If you receive someone’s blood, will it make you like them? | 19 | 81 |
| Do you believe blood can be manufactured? | 17 | 83 |
| Do you believe that receiving a blood donation could help to save a person’s life? | 91 | 9 |
| When people need blood, do you believe they must be made to pay for it? | 56 | 44 |
| Do you believe it is legal in Sierra Leone for a person to be paid for giving blood? | 45 | 55 |
| Do you believe that donating blood can make you infertile? | 22 | 78 |
| Is there an age limit to donating blood? | 71 | 29 |
| Can pregnant women donate blood? | 10 | 90 |
| Can lactating mothers donate blood? | 18 | 82 |
| Can women donate blood when menstruating (monthly bleeding)? | 17 | 83 |
| Can smokers donate blood? | 61 | 39 |
| Can people with tattoos donate blood? | 50 | 50 |
| Have you ever bought blood for your family and/or relatives? | 30 | 70 |
| Have you ever donated blood as a volunteer to the blood bank? | 20 | 80 |

**Table S2. Donor survey of motivations**

| **Survey item** | **n** | **%** |
| --- | --- | --- |
| I get food at the blood drive | 29 | 35 |
| I like being admired in my community | 16 | 19 |
| I want to save lives | 60 | 71 |
| I feel pressurised by the organisational team | 14 | 17 |
| I know God is pleased with me | 19 | 23 |
| It’s a fun time with other people | 7 | 8 |
| If I need blood or my family member needs blood one day, we  will get it | 41 | 49 |
| I feel pressurised by my friends | 1 | <1 |
| I get my blood checked for HIV and other diseases | 40 | 48 |
| I enjoy being known as a blood donor | 44 | 52 |
| Another reason. Please write below: |  |  |
| For my family and for myself | 4 | 5 |
| To reduce my blood | 1 | <1 |
| To help my fellow human beings | 1 | <1 |
| To get good health | 3 | 4 |
| I don’t want to buy blood again | 1 | <1 |
| As a nurse, I do it for the pregnant women | 1 | <1 |

**Table S3. Costs associated with different phases of the project cycle**

| **Stage of project** | **Cost** | **Cost per drive** | **Cost per unit** |
| --- | --- | --- | --- |
| Non-blood drive community engagement/involvement activities & set-up expenses | 127,99  (£5,818) |  |  |
| Drives for months 3-7 |  | 30,712 Nle  (£1,396) | 389 Nle  (£18) |
| Sustainable period  (months 8-10) |  | 10,604 Nle  £482 | 131 Nle  (£6) |
| Projected ongoing cost if funds  available |  | 9,436 Nle  (£429) | 18 Nle  (£5.36) |
